# Design and synthesis of cysteine-specific labels for photo-crosslinking studies†

**DOI:** 10.1039/c8ra10436k

**Published:** 2019-03-07

**Authors:** Martin Walko, Eric Hewitt, Sheena E. Radford, Andrew J. Wilson

**Affiliations:** School of Chemistry, University of Leeds Leeds LS2 9JT UK a.j.wilson@leeds.ac.uk; Astbury Centre for Structural Molecular Biology, University of Leeds Leeds LS2 9JT UK; School of Molecular and Cellular Biology, Faculty of Biological Sciences, University of Leeds Leeds LS2 9JT UK

## Abstract

Chemical cross-linking mass-spectrometry (XL-MS) represents a powerful methodology to map ligand/biomacromolecule interactions, particularly where conventional methods such as X-ray crystallography, nuclear magnetic resonance (NMR) spectroscopy or cryo-electron microscopy (EM) are not feasible. In this manuscript, we describe the design and synthesis of two new photo-crosslinking reagents that can be used to specifically label free thiols through either maleimido or methanethiosulfonate groups and facilitate PXL-MS workflows. Both crosslinkers are based on light sensitive diazirines – precursors of highly reactive carbenes which offer additional advantages over alternative crosslinking groups such as benzophenones and aryl nitrenes given the controlled rapid and more indiscriminate reactivity.

## Abstract 2

The design and synthesis of cysteine specific diazirine containing labels is described.

## Graphical abstract

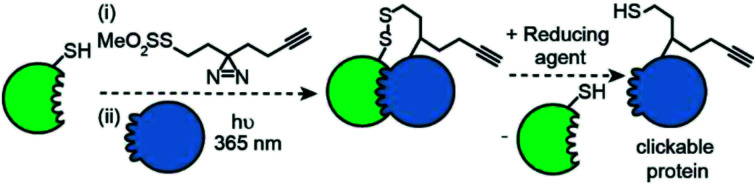

## Introduction

**Fig. 1:**
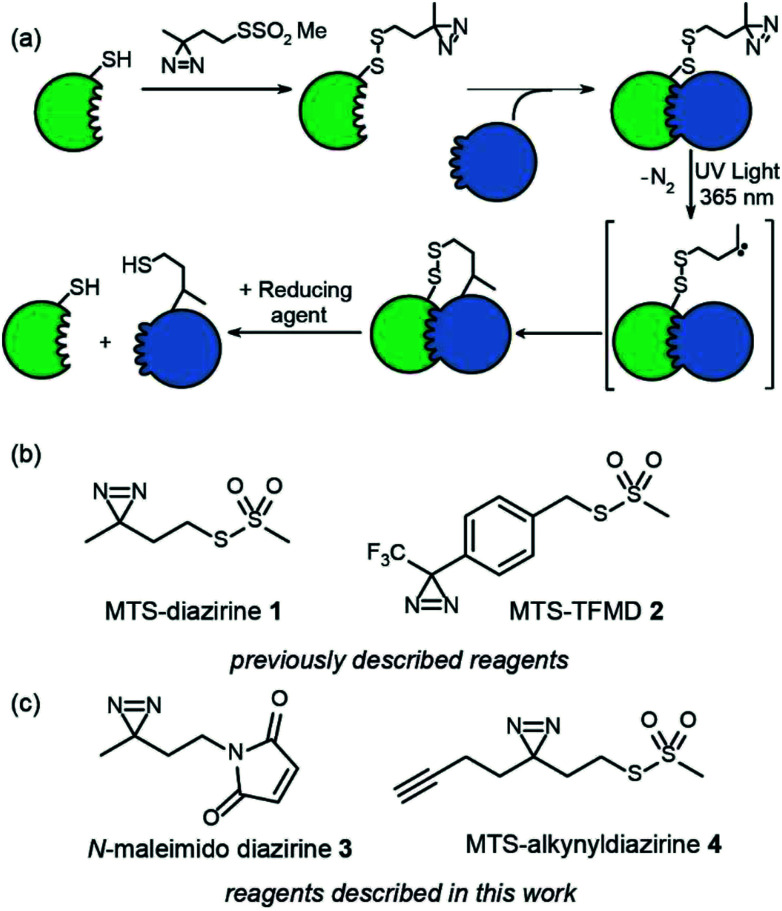
Overview of tag-transfer PXL for mapping PPIs and diazirine based PXL reagents, (a) tag-transfer PXL workflow: a thiol-containing “bait” protein (green) is labelled with the reagent (here MTS-diazirine). After adding a partner protein (blue), irradiation with 365 nm UV light, reveals a carbene that can react with the partner protein. Reduction of the disulphide reveals a thiol which can be further labelled, (b) previously described diazirine based PXL reagents (c) diazirine based PXL reagents described in this study.

Chemical crosslinking-mass spectrometry (XL-MS) is an increasingly important approach to map biomacromolecule and small-molecule/biomacromolecule interactions.^1–4^ Furthermore, XL-MS is uniquely placed to facilitate analysis of transient and dynamic interactions and/or conformational changes. Given that cross-linking “covalently traps” a protein or its complex, XL-MS has the potential to map changes in conformation and interactions with time. However, to do so requires reactive intermediates that react rapidly and indiscriminately with proximal functionality to ensure supramolecular connectivity is accurately captured upon cross-linking.^5^ In this regard photoinduced cross-linking (PXL)^6^ is advantageous in that light is used to trigger cross-linking and the suite of functionalities used for PXL tend to be more indiscriminate in their reactivity than traditional cross-linking reagents^7–9^ such as NHS-esters which require nucleophilic residues to be present at an interface. We recently synthesized two cysteine specific diazirines as PXL reagents and outlined a workflow for mapping protein interactions that exploits tag-and-transfer (Fig. 1a and b).^10^ Using a cysteine selective methanethiosulfonate (MTS) or maleimido group to attach the reagents to the protein allows precise positioning of the crosslinking functionality within the protein. The photosensitive diazirines represent ideal functional groups for PXL-MS workflows^5,11–17^ – upon irradiation, the resultant carbenes insert rapidly into X–H bonds (including O–H, N–H, S–H and C–H bonds).^10^ Diazirines have been shown to be advantageous over other common cross-linking groups (*e.g.* benzophenone and phenyl azide) arising as a consequence of more rapid and indiscriminate reactivity leading to effective encoding of supramolecular connectivity.^18^ However the synthesis of dizarines is more challenging, limiting their use.

In our prior work, we introduced two heterobifunctional reagents (MTS-diazirine 1 and MTS-TFMD 2) (Fig. 1b) bearing a methanethiosulphonate group to facilitate specific labelling of Cys residues on a “bait” protein, creating a cleavable disulfide bond bearing a diazirine.^10^ Following cross-linking with partner proteins, this disulfide could be reduced leaving a thiol on the partner protein. In this work we describe the design and synthesis of two further reagents; (i) *N*-maleimido-diazirine 3 and (ii) MTS-alkynyldiazirine 4 (Fig. 1c). *N*-Maleimido-diazirine 3 can be used to label thiols on peptides or proteins *via* a conventional conjugate addition, used widely in protein labelling.^19^ MTS-alkynyldiazirine 4 can be used to label thiols on peptides or proteins, yet bears an additional bio-orthogonal group (the alkyne) that could be exploited to introduce further functionality (*e.g.* fluorophore, biotin) by “click” chemistry^20,21^ to support chemical proteomics workflows.

## Results and discussion

**Fig. 2:**
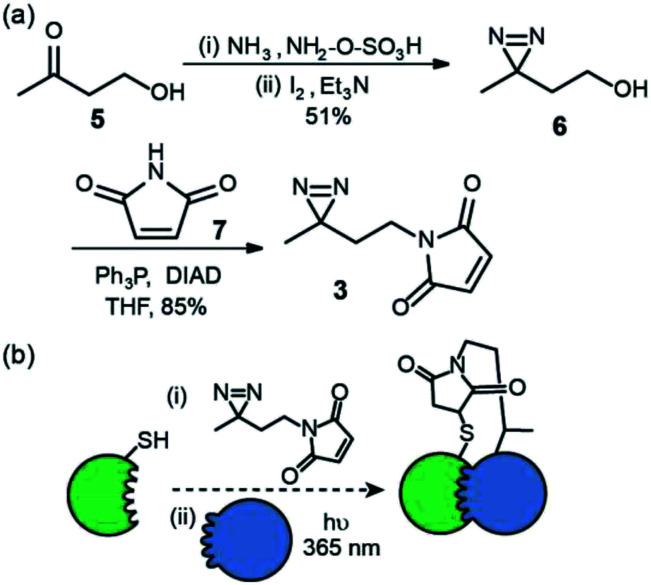
Synthesis and use of *N*-maleimido diazirine (a) synthesis (b) utility for PXL workflows; a thiol on a protein can be used to react with the *N*-maleimide following which introduction of partner proteins and excitation generates a permanent cross-link *via* the reactive carbene.

We first prepared an alkyl diazirine based reagent that could be used to label proteins *via* Michael addition of a thiol to a maleimide. This bioconjugation reaction is widely used^19^ and trivial to perform for non-specialists whilst avoids the use of more hazardous and less chemoselective alkylating reagents *e.g.* α-halocarbonyl containing or iodoalkyl reagents. *N*-Maleimido-diazirine 3 was prepared in two steps (Fig. 2a) from 4-hydroxybutan-2-one 5. One pot introduction of the diazirine group to give compound 6 ^22^ was achieved *via* iodine mediated oxidation of the diaziridine that results from reaction of the ketone hydroxylamine-*O*-sulfonic acid in liquid ammonia. This was followed by Mitsunobu reaction with *N*-maleimide 7, to yield the labelling reagent in 43% over two steps.

This reagent could in principle be used to label thiols on proteins and exploited in PXL-MS (Fig. 2b). A difference in using this reagent in contrast to the tag-and-transfer reagents described previously is that following cross-linking, a permanent covalent link between bait and partner proteins will be generated, which may be advantageous under conditions where the former reagents are unstable (*e.g.* the reducing environment of the cell).

**Fig. 3:**
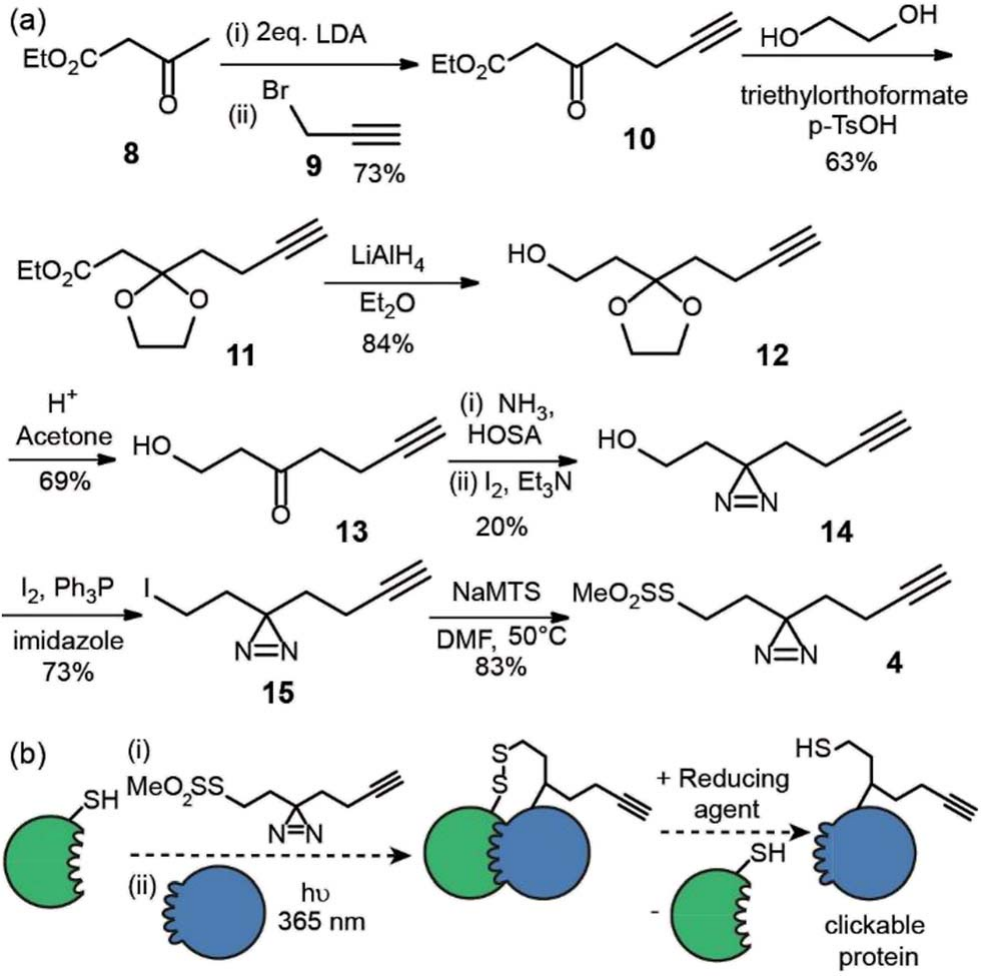
Synthesis and use of MTS-alkynyldiazirine (a) synthesis (b) utility for PXL workflows; a thiol on a protein can be used to displace the MTS group following which, introduction of partner proteins and excitation generates a cross-link *via* the reactive carbene, then the disulphide can be reduced resulting in the transfer of a thiol and biorthogonal alkyne onto the partner protein.

The second reagent has been designed to be used as a tag-and-transfer diazirine, but with an additional biorthogonal alkyne. Consequently, the synthesis was longer, however gram quantities could be prepared. Beginning with ethyl acetoacetate 8, alkylation with 3-bromopropyne 9 and protection of the ketone 10 as the cyclic acetal 11, followed by reduction of the ester to alcohol 12 ^23^ and removal of the acetal afforded the hydroxyl ketone substrate 13 for diazirine ring formation. As before, the reaction of the ketone with hydroxylamine-*O*-sulfonic acid in liquid ammonia followed by oxidation with iodine gave diazirine 14.^24^ Subsequent conversion of the alcohol 14 to an alkyl iodide 15 and reaction with sodium methanethiosulfonate afforded the final tag-transfer MTS-alkynyldiazirine 4 (Fig. 3).

This label could be used in tag and transfer PXL-MS applications in the same way as our previously described workflow.^10^ Labelling of bait protein followed by photocrosslinking and reduction, transfers onto a partner protein a thiol group, but also an alkyne. We envision this second cysteine-specific label as being of use for the enrichment of cross-linked proteins where multiple thiols are already present in the partner protein or other components of the sample under analyses; the biorthogonal advantages of the alkyne should be readily exploited. Alternatively, the alkyne could be used to ligate *e.g.* fluorophores for the construction of biosensors.^25^

## Conclusions

In summary, we have described the design and synthesis of two new diazirine based cross-linking reagents that can be used to specifically label free thiols. These labels could be applied in structural analyses of protein–protein interactions using PXL-MS by conjugating them to bait peptides or proteins and subsequent crosslinking with partner proteins. In principle, such a strategy could readily be applied to other ligand/biomacrolecule interactions as long as a thiol is present in the bait ligand.

## Experimental section

### General considerations

All solvents were purchased from Fisher Scientific and all reagents were purchased from Sigma-Aldrich or Fluorochem unless otherwise stated and used without further purification. Purification by column chromatography was carried out using silica gel. Analytical thin layer chromatography (TLC) was conducted using Merck 0.25 mm silica gel pre-coated aluminium plates with fluorescent indicator active at UV245. ^1^H and ^13^CNMR spectra were acquired on Bruker Avance III HD 400 series spectrometer at 400 MHz for ^1^H, and 100 MHz for ^13^C. Chemical shifts are expressed as parts per million using solvent as internal standard (CDCl_3_ 7.26 ppm in ^1^H and 77.16 ppm in ^13^C spectra) and coupling constants are expressed in Hz. The following abbreviations are used: s for singlet, d for doublet, t for triplet, q for quartet, m for multiplet and br for broad.

### 2-(3-Methyl-3*H*-diazirin-3-yl)ethanol (6)^22^

NH_3_ (approx. 100 mL) was condensed, using a dry ice/acetone condenser, into a flask containing 4-hydroxy-2-butanone 5 (15.2 mL, 170 mmol) and cooled to −78 °C. After refluxing (−30 °C bath temperature) for 5 h, hydroxylamine-*O*-sulfonic acid (21.15 g, 187 mmol) dissolved in MeOH (150 mL) was added at −78 °C and the reaction mixture was allowed to heat to room temperature overnight. The resulting mixture was filtered, the solid residue was washed with MeOH (2 × 20 mL), and the filtrate was concentrated to about 100 mL. Triethylamine (26 mL, 187 mmol) was added to the resulting solution followed by iodine in several portions while cooling the reaction mixture in ice. After adding 29.2 g (115 mmol) of I_2_, the colour of iodine persisted, indicating the end of the reaction. The solvents were carefully removed from the reaction mixture (25 °C and 120 mbar) and the residue was partitioned between Et_2_O (200 mL) and brine (200 mL) containing sat. aq. Na_2_S_2_O_3_ (10 mL). The organic layer was separated and the aqueous layer was extracted with Et_2_O (2 × 100 mL). The combined organic extracts were dried over Na_2_SO_4_ and concentrated to give crude diazirine 6, which was purified by column chromatography (SiO_2_, pentane/Et_2_O 1/1) to give 8.64 g (51%) of product as a colourless liquid. *δ*_H_ (400 MHz, CDCl_3_) 1.08 (s, 3*H*, CH_3_), 1.45 (brs, 1*H*, OH), 1.64 (t, 2*H*, *J* = 6.3 Hz, CH_2_), 3.54 (t, 2*H*, *J* = 6.3 Hz, CH_2_).

### 1-(2-(3-Methyl-3*H*-diazirin-3-yl)ethyl)-1*H*-pyrrole-2,5-dione (3)

Diisopropyl azodicarboxylate (2.4 mL, 12 mmol) was added dropwise to a solution of alcohol 6 (1 g, 10 mmol), Ph_3_P (2.885 g, 11 mmol) and maleimide 7 (1.067 g, 11 mmol) in THF (30 mL) at 0 °C. After stirring the mixture for 16 h at room temperature, the solvent was evaporated and the residue was triturated with mixture of 15 mL Et_2_O and 15 mL of hexane and filtered. The filtrate was concentrated and purified by column chromatography (SiO_2_, hexane/EtOAc 4/1 to 2/1) to give 1.52 g (85%) of product 3 as a colourless oil. *δ*_H_ (400 MHz, CDCl_3_) 1.06 (s, 3*H*, CH_3_CN_2_), 1.60 (t, 2*H*, *J* = 7.1 Hz, CH_2_CN_2_), 3.57 (d, 2*H*, *J* = 7.1 Hz, CH_2_N), 6.73 (s, 2*H*, CH = CH); *δ*_C_ (100 MHz, CDCl_3_) 19.28, 24.04, 33.33, 33.51, 134.41, 170.59; ESI-HRMS found *m*/*z* 202.0575 [M + Na]^+^ C_8_H_9_N_3_NaO_2_ requires 202.0592. IR: *ν*_max_/cm^−1^ (oil) = 1699, 1593, 1443, 1406, 1387, 1364, 1310, 825, 693; UV-vis: *λ*_max_(*ε*) (CH_3_CN) = 216 (14 100), 301 (607), 360sh (60).

### Ethyl 3-oxohept-6-ynoate (10)^23^

*n*-BuLi (100 mL of 1.6 M solution in hexanes, 160 mmol) was added to a solution of *N*,*N*-diisopropylethylamine (22.5 mL, 160 mmol) in dry THF (150 mL) at 0 °C under N_2_. After 30 min of stirring, ethyl acetoacetate 8 (10.1 mL, 80 mmol) was slowly added and after another 30 min propargyl bromide 9 (7.1 mL, 80% solution in toluene, 80 mmol) was added to the reaction mixture at 0 °C under N_2_. After stirring for additional 1 h under the same conditions, AcOH (9.2 mL, 160 mmol) was added to quench the reaction, followed by water (100 mL). The organic layer was separated and water layer was extracted with ether (2 × 100 mL). The combined organic layers were washed with brine (100 mL), dried over MgSO_4_ and concentrated *in vacuo*. Target material was purified by column chromatography (SiO_2_, hexane/ethyl acetate 4/1 to 2/1) to give 9.81 g (73%) of product as a colourless oil. *δ*_H_ (400 MHz, CDCl_3_) 1.26 (t, 3*H*, *J* = 7.2 Hz, CH_3_), 1.94 (t, 1*H*, *J* = 2.6 Hz, C≡CH), 2.45 (dt, 2*H*, *J* = 7.2, 2.6 Hz, CH_2_C≡C), 2.79 (t, 1*H*, *J* = 7.2 Hz, CH_2_CH̲_2_CO), 3.44 (s, 2*H*, CH̲_2_COOEt), 4.18 (q, 2*H*, *J* = 7.2 Hz, CH̲_2_CH_3_).

### Ethyl 2-(2-(but-3-yn-1-yl)-1,3-dioxolan-2-yl)acetate (11)^23^

Ethyl 3-oxohept-6-ynoate 10 (9.50 g, 56.5 mmol) was dissolved in the mixture of ethylene glycol (12.6 mL, 226 mmol) and triethyl orthoformate (18.8 mL, 113 mmol). *p*-Toluenesulfonic acid monohydrate (1.08 g, 5.7 mmol) was added and the resulting solution was stirred at rt for 16 h, then sat. aq. NaHCO_3_ (50 mL) was added and the mixture was extracted with ether (3 × 100 mL). The combined organic layers were washed with brine (100 mL), dried over MgSO_4_ and concentrated *in vacuo*. Target material was purified by column chromatography (SiO_2_, hexane/ethyl acetate 4/1 to 2/1) to give 7.56 g (63%) of product as a colourless oil. *δ*_H_ (400 MHz, CDCl_3_) 1.26 (t, 3*H*, *J* = 7.2 Hz, CH_3_), 1.92 (t, 1*H*, *J* = 2.6 Hz, C≡CH), 2.11 (t, 2*H*, *J* = 7.9 Hz, CH̲_2_CH_2_C≡C), 2.27–2.32 (m, 2*H*, CH_2_C≡C), 2.64 (s, 2*H*, CH̲_2_COOEt), 3.94–4.03 (m, 4*H*, OCH_2_CH_2_O), 4.15 (q, 2*H*, *J* = 7.1 Hz, CH̲_2_CH_3_).

### 2-(2-(But-3-yn-1-yl)-1,3-dioxolan-2-yl)ethanol (12)^23^

Ethyl 2-(2-(but-3-yn-1-yl)-1,3-dioxolan-2-yl)acetate 11 (7.5 g, 35.3 mmol) in dry ether (20 mL) was added to a mixture of LiAlH_4_ (1.47 g, 38.8 mmol) in dry ether (200 mL) at 0 °C. The reaction mixture was stirred for 2 h at room temperature and then slowly quenched with water (100 mL). The organic layer was separated and water layer was extracted with ether (2 × 100 mL). The combined organic layers were washed with brine (100 mL), dried over MgSO_4_ and concentrated *in vacuo*. The resulting material was purified by column chromatography (SiO_2_, hexane/ethyl acetate 4/1) to give 5.07 g (84%) of product as a colourless oil. *δ*_H_ (400 MHz, CDCl_3_) 1.90–1.95 (m, 5*H*, 2× CH_2_, C≡CH), 2.23–2.29 (m, 2*H*, CH_2_C≡C), 2.66 (brs, 1*H*, OH), 3.71–3.77 (m, 2*H*, CH̲_2_OH), 3.94–4.03 (m, 4*H*, OCH_2_CH_2_O).

### 1-Hydroxyhept-6-yn-3-one (13)^24^

2-(2-(But-3-yn-1-yl)-1,3-dioxolan-2-yl)ethanol 12 (5.00 g, 29.4 mmol) was dissolved in acetone (50 mL), *p*-toluenesulfonic acid monohydrate (0.288 g, 1.5 mmol) was added and the resulting solution was stirred at rt for 3 h. Sat. aq. NaHCO_3_ (50 mL) was added to the reaction mixture which was subsequently extracted with ethyl acetate (3 × 50 mL). The combined organic layers were washed with brine (50 ml), dried over MgSO_4_ and concentrated *in vacuo*. The target material was purified by column chromatography (SiO_2_, hexane/ethyl acetate 1/1 to pure ethylacetate) to give 2.56 g (69%) of product as a colourless oil; (400 MHz, CDCl_3_) 1.95 (t, 1*H*, *J* = 2.7 Hz, C≡CH), 2.45 (td, 2*H*, *J* = 7.4, 2.7 Hz, CH_2_C≡C), 2.46–2.50 (m, 1*H*, OH), 2.67–2.70 (m, 4*H*, 2× CH_2_), 3.85 (q, 2*H*, *J* = 5.7 Hz, CH̲_2_OH).

### 2-(3-(But-3-yn-1-yl)-3*H*-diazirin-3-yl)ethanol (14)^24^

NH_3_ (approx. 50 mL) was condensed, using dry ice/acetone condenser, into a flask containing 1-hydroxyhept-6-yn-3-one 13 (2.50 g, 19.8 mmol) and cooled to −78 °C. After refluxing (−30 °C bath temperature) for 5 h, hydroxylamine-*O*-sulfonic acid (2.47 g, 21.8 mmol) dissolved in MeOH (20 mL) was added at −78 °C and the reaction mixture was allowed to heat to room temperature overnight. Resulting mixture was filtered, solid residue was washed with MeOH (2 × 20 mL), and filtrate was concentrated to about 20 mL. Triethylamine (3 mL, 21.8 mmol) was added to the resulting solution followed by iodine in several portions while cooling the reaction mixture in ice. After adding 2.06 g (8.1 mmol) of I_2_, the colour of iodine persisted, indicating the end of the reaction. The solvents were removed from the reaction mixture and residue was partitioned between Et_2_O (50 mL) and brine (50 mL) containing sat. aq. Na_2_S_2_O_3_ (5 mL). The organic layer was separated and the aqueous layer was extracted with Et_2_O (2 × 50 mL). The combined organic extracts were dried over MgSO_4_ and concentrated to give crude diazirine 14, which was purified by column chromatography (SiO_2_, pentane/Et_2_O 1/1) to give 0.528 g (20%) of product as a colourless liquid. *δ*_H_ (400 MHz, CDCl_3_) 1.57 (brs, 1*H*, OH), 1.68 (t, 2*H*, *J* = 7.4 Hz, CH̲_2_CH_2_C≡C), 1.71 (t, 2*H*, *J* = 6.2 Hz CH̲_2_CH_2_OH), 2.00 (t, 1*H*, *J* = 2.6 Hz, C≡CH), 2.04 (td, 2*H*, *J* = 7.4; 2.5 Hz, Hz, CH_2_C≡C), 3.49 (t, 2*H*, *J* = 6.2 Hz, CH̲_2_OH)

### 3-(But-3-yn-1-yl)-3-(2-iodoethyl)-3*H*-diazirine (15)^25^

Iodine (305 mg, 1.2 mmol) was added to a solution of Ph_3_P (315 mg, 1.2 mmol) and imidazole (163 mg, 2.4 mmol) in dichloromethane (5 mL) at 0 °C. After 15 min stirring, the alcohol 14 (138 mg, 1.0 mmol) in dichloromethane (1 mL) was added and the mixture was stirred for 4 h at room temperature. Water (10 mL) was added to the reaction mixture, organic layer was separated and aqueous layer was extracted with dichloromethane (2 × 10 mL). The combined organic extracts were dried over MgSO_4_ and concentrated. The residue was purified by column chromatography (SiO_2_, hexane/Et_2_O 10/1) to give 180 mg (73%) of product as a colourless oil. *δ*_H_ (400 MHz, CDCl_3_) 1.67 (t, 2*H*, *J* = 7.2 Hz, CH̲_2_CH_2_C≡C), 1.99–2.04 (m, 3*H*, CH_2_C≡CH), 2.11 (t, 2*H*, *J* = 7.6 Hz, CH̲_2_CH_2_I), 2.88 (t, 2*H*, *J* = 7.6 Hz, CH̲_2_I); *δ*_C_ (100 MHz, CDCl_3_) −3.87, 13.37, 28.79, 31.94, 37.63, 69.57, 82.53.

### *O*-Methyl 2-(3-(but-3-yn-1-yl)-3*H*-diazirin-3-yl)ethanesulfono-thioate (4)

Sodium methanethiosulfonate (117 mg, 0.87 mmol) was added to a solution of iodide 15 (180 mg, 0.73 mmol) in DMF (1 mL) and the resulting solution was heated at 50 °C for 4 h. After evaporation of the solvent, the residue was purified by column chromatography (SiO_2_, hexane/ethyl acetate 3/1) to give 140 mg (83%) of product as colourless oil. *δ*_H_ (400 MHz, CDCl_3_) 1.66 (t, 2*H*, *J* = 7.2 Hz, CH̲_2_CH_2_C≡C), 1.92 (t, 2*H*, *J* = 7.6 Hz, CH̲_2_CH_2_SSO_2_), 1.99–2.04 (m, 3*H*, CH_2_C≡CH), 2.95 (t, 2*H*, *J* = 7.6 Hz, CH_2_SSO_2_), 3.31 (s, 3*H*, CH_3_SO_2_); *δ*_C_ (100 MHz, CDCl_3_) 13.25, 27.35, 30.41, 32.03, 33.46, 50.67, 69.71, 82.19. ESI-HRMS found *m*/*z* 255.0226 [M + Na]^+^ C_8_H_12_N_2_NaO_2_S_2_ expected 255.0238; IR: *ν*_max_/cm^−1^ (solid state) = 3288, 1588, 1434, 1312, 1128, 954, 743; UV-vis: *λ*_max_(*ε*) (CH_3_CN) = 346 (51), 360sh (41).

## Conflicts of interest

There are no conflicts to declare.

## Supplementary Material

RA-009-C8RA10436K-s001
