# Supplementary material for: Design and synthesis of cysteine-specific labels for photo-crosslinking studies
Source: RSC Adv. Author manuscript; Available in PMC 2025 May 22. (PMC9061181; doi:10.1039/c8ra10436k)

Electronic Supplementary Material (ESI) for RSC Advances.
This journal is © The Royal Society of Chemistry 2019

Supporting information

Design and Synthesis of Cysteine-Specific Labels for Photo-Crosslinking Studies

Martin Walko,[a,b] Eric Hewitt,[b,c] Sheena E. Radford,[b,c] and Andrew J Wilson,*[a,b]

[a] School of Chemistry, University of Leeds, Leeds, LS2 9JT (UK) E-mail: a.j.wilson@leeds.ac.uk

[b] Astbury Centre for Structural Molecular Biology, University of Leeds, Leeds, LS2 9JT (UK).

[c] School of Molecular and Cellular Biology, Faculty of Biological Sciences, University of Leeds, Leeds, LS2 9JT (UK).

^{1}H NMR of 2-(3-Methyl-3H-diaziren-3-yl)ethanol (**6**)

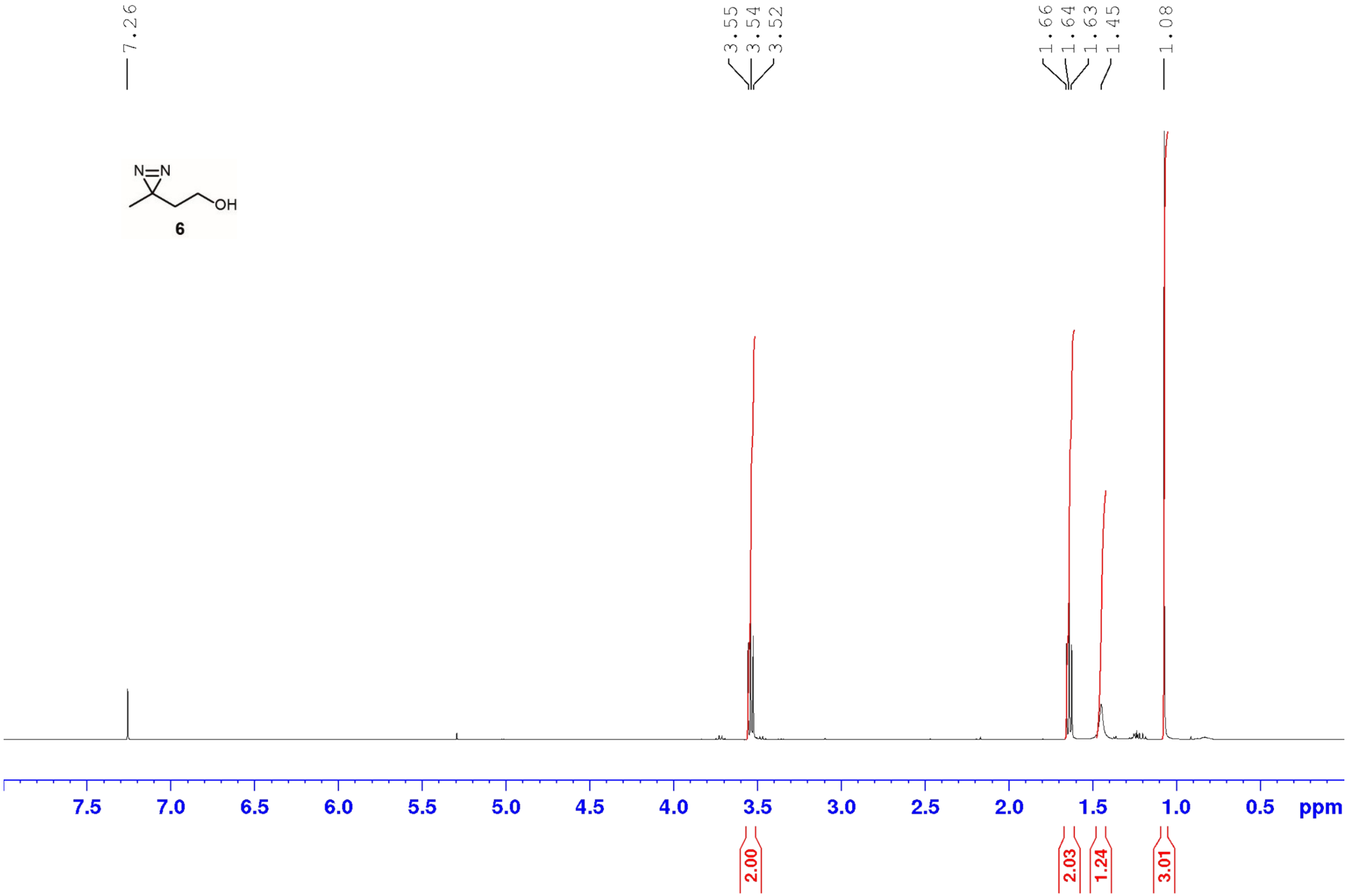

^{1}H NMR of 1-(2-(3-methyl-3H-diazirin-3-yl)ethyl)-1H-pyrrole-2,5-dione (**3**)

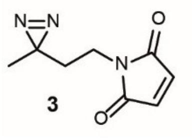

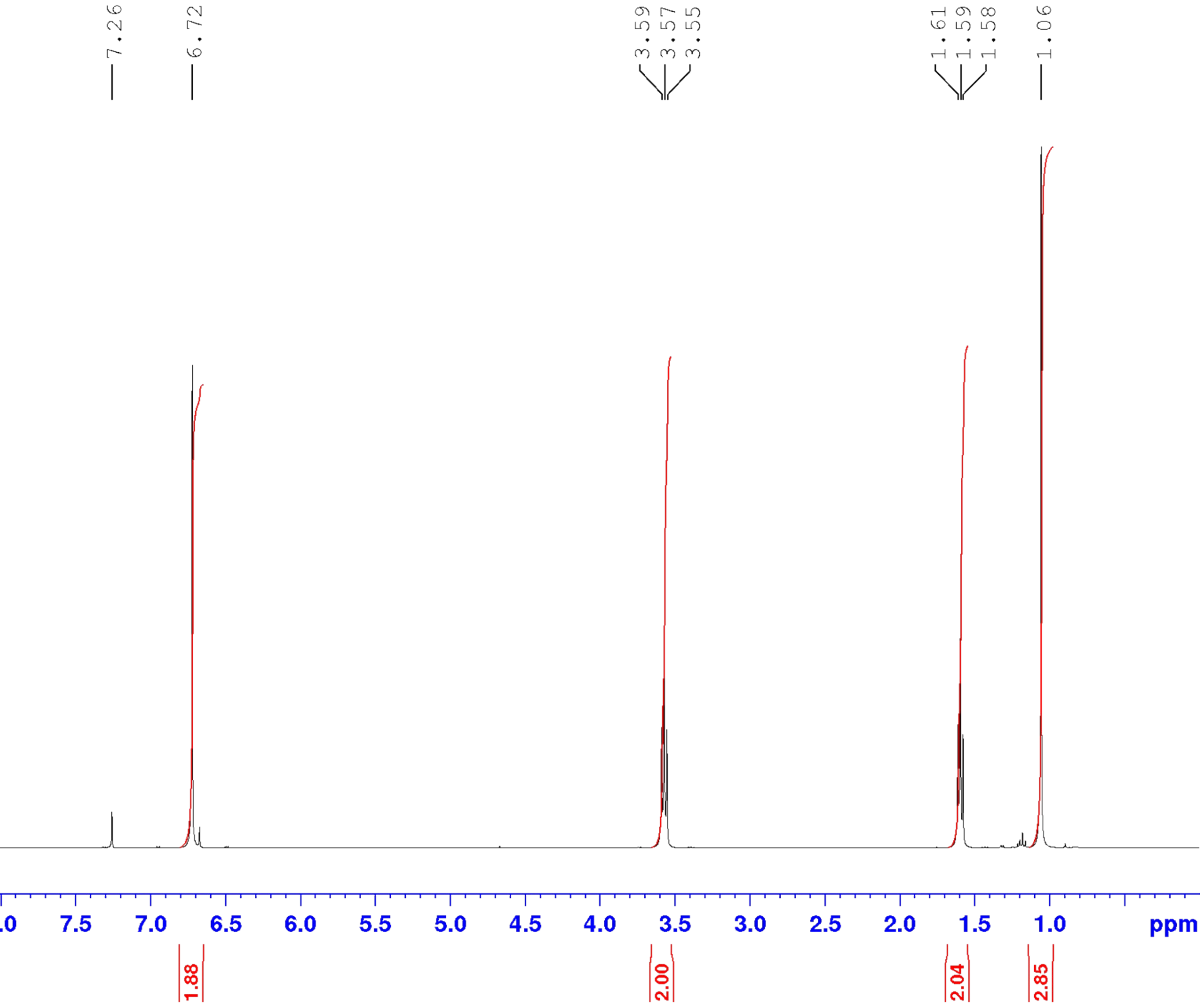

^{13}C NMR of 1-(2-(3-methyl-3H-diazirin-3-yl)ethyl)-1H-pyrrole-2,5-dione (**3**)

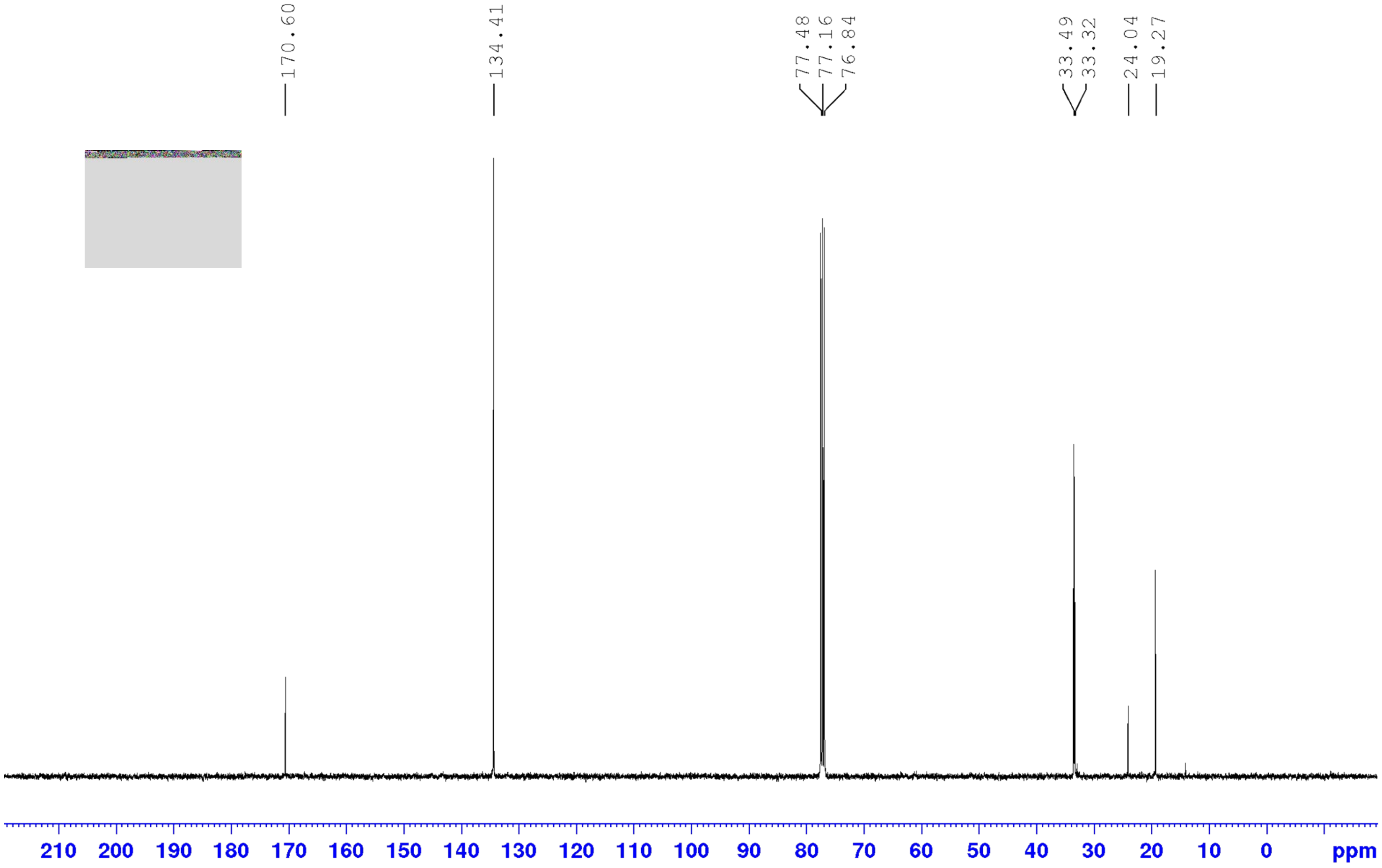

UV-vis of 1-(2-(3-methyl-3H-diazirin-3-yl)ethyl)-1H-pyrrole-2,5-dione (**3**)

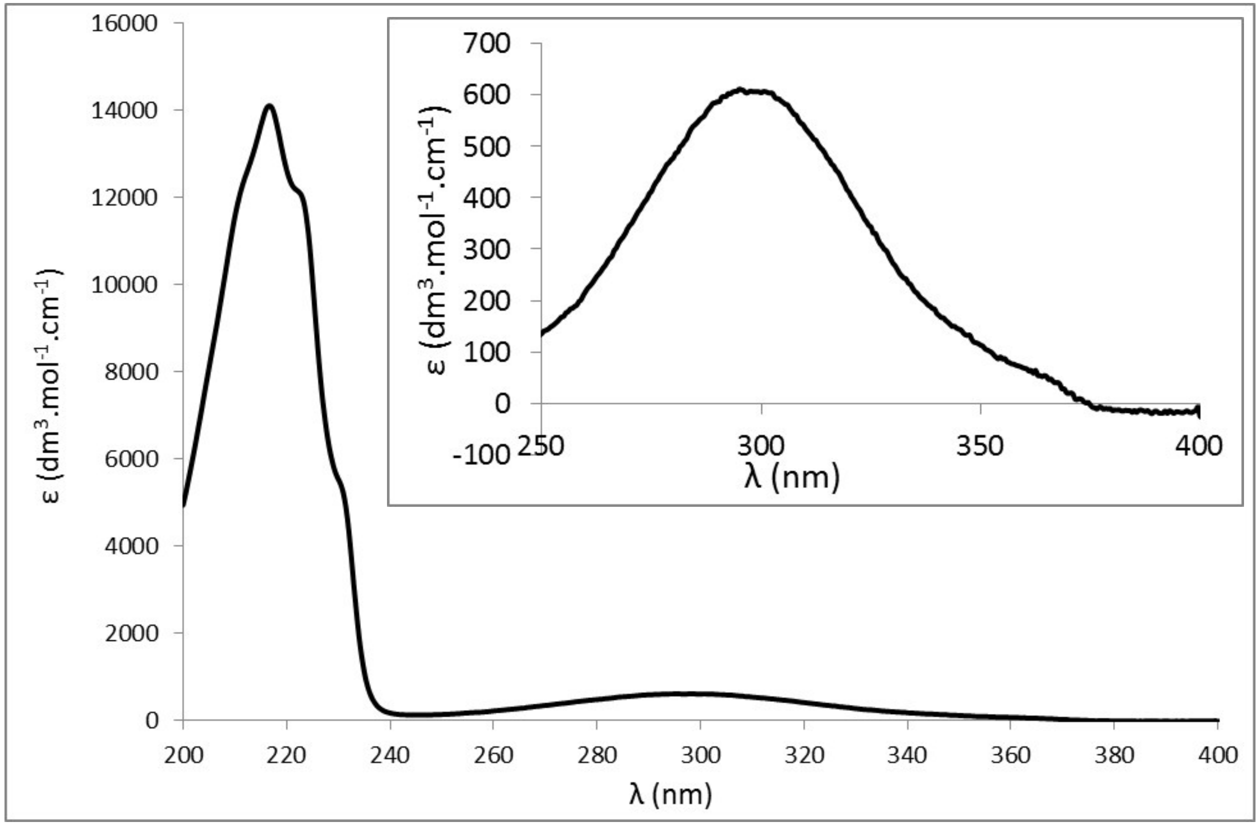

FT-IR of 1-(2-(3-methyl-3H-diazirin-3-yl)ethyl)-1H-pyrrole-2,5-dione (**3**)

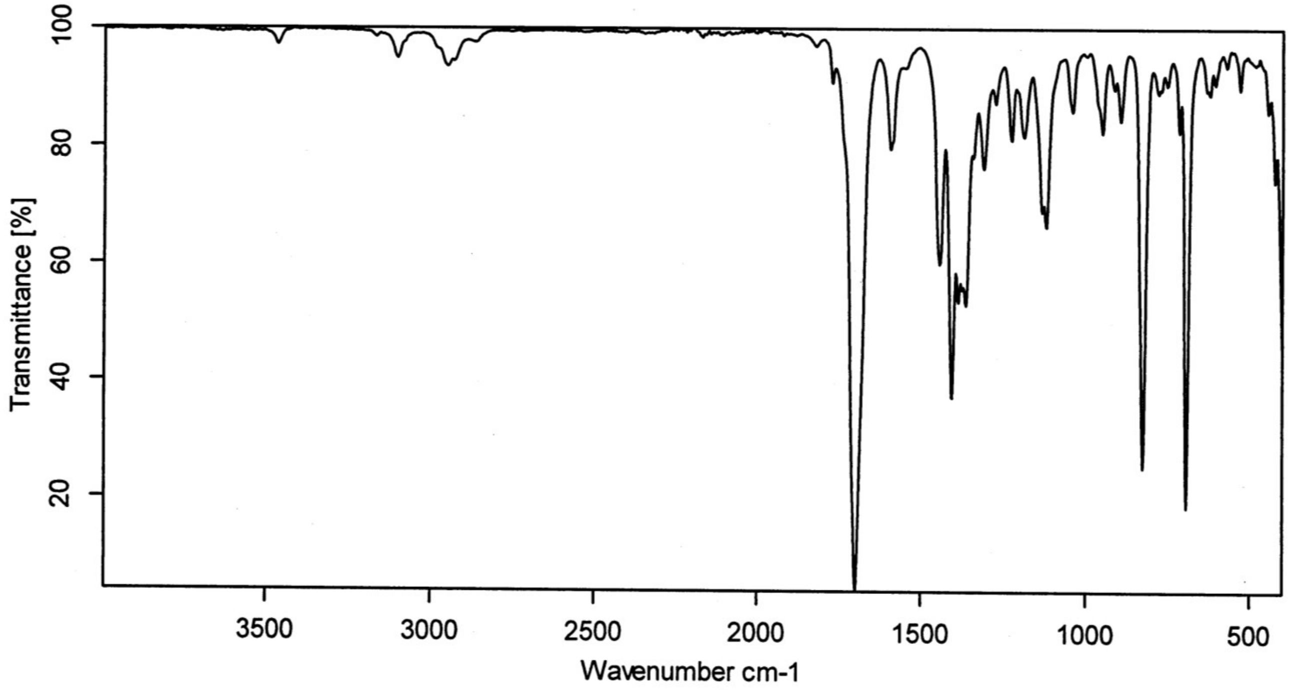

^{1}H NMR of ethyl 3-oxohept-6-ynoate (**10**)

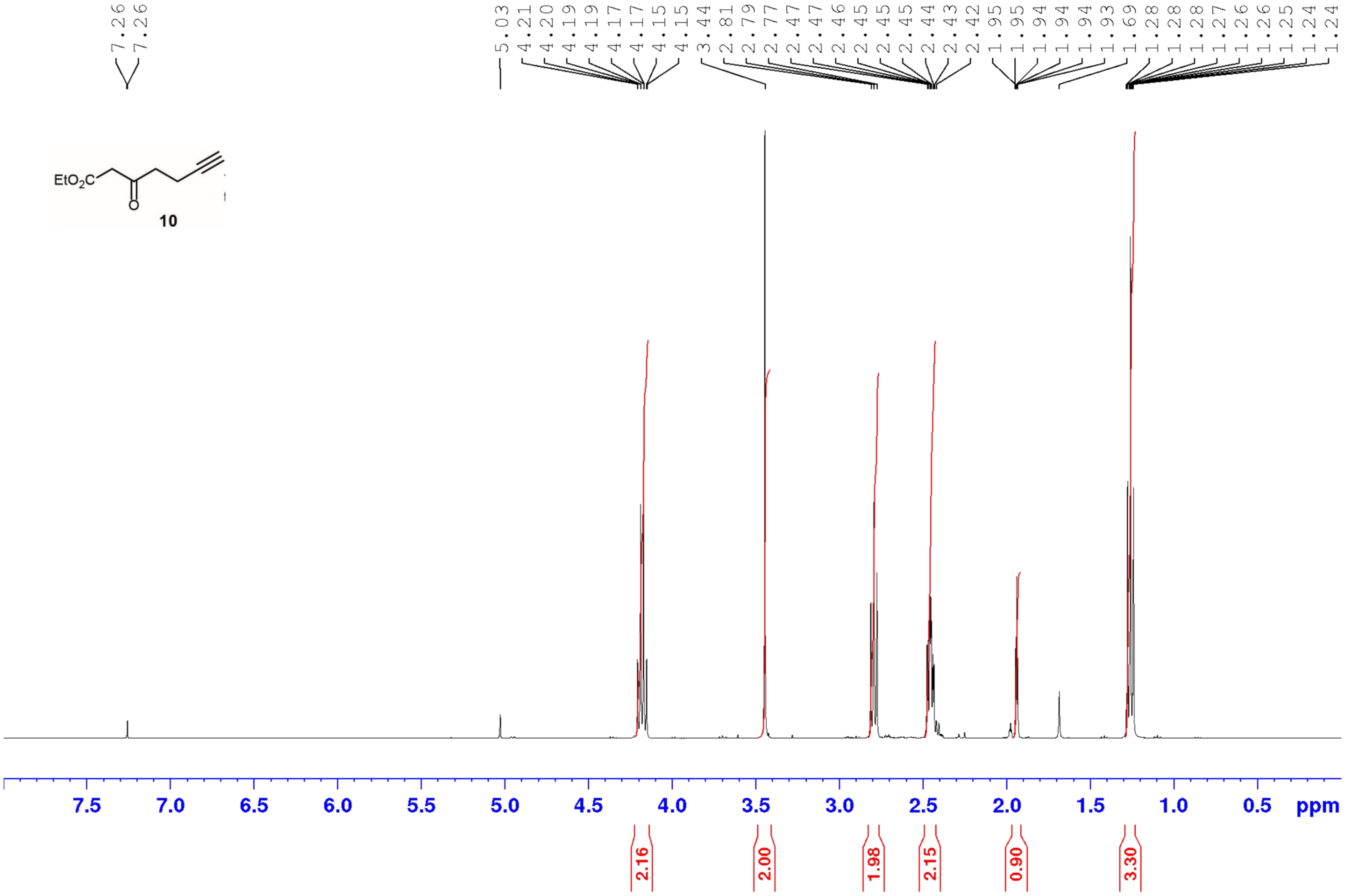

^{1}H NMR of ethyl 2-(2-(but-3-yn-1-yl)-1,3-dioxolan-2-yl)acetate (**11**)

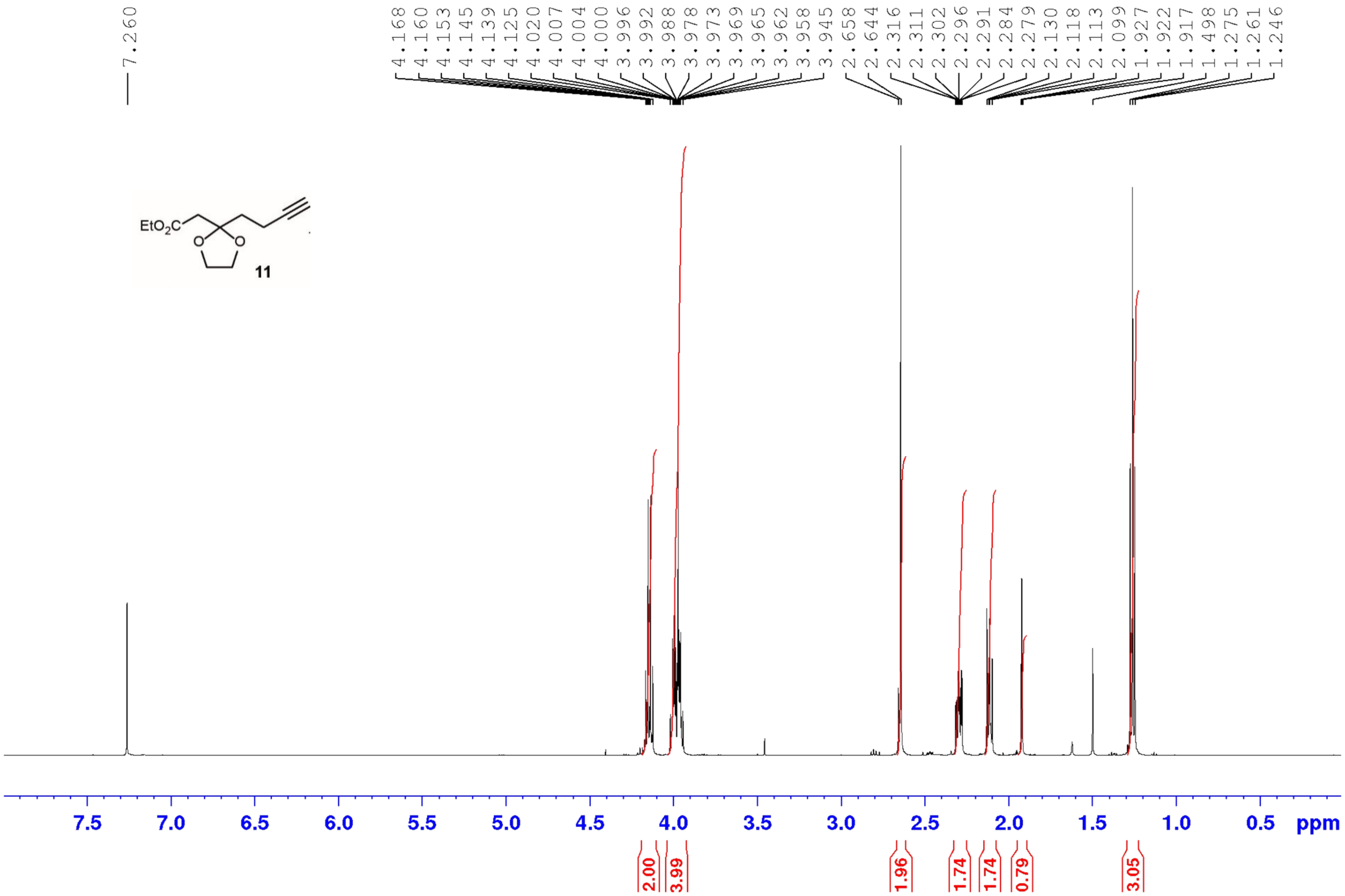

^{1}H NMR of 2-(2-(but-3-yn-1-yl)-1,3-dioxolan-2-yl)ethanol (**12**)

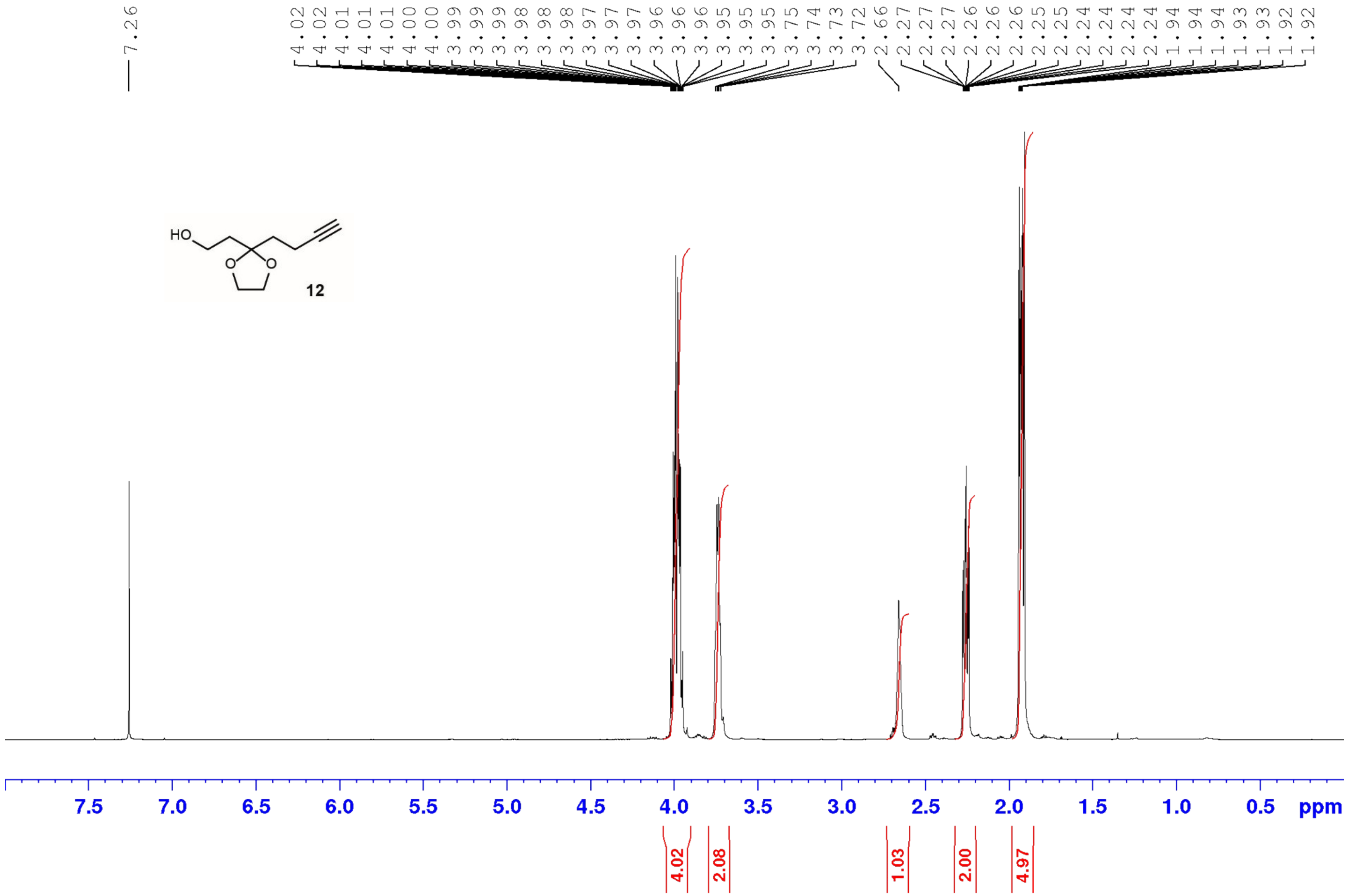

^{1}H NMR of 1-hydroxyhept-6-yn-3-one (**13**)

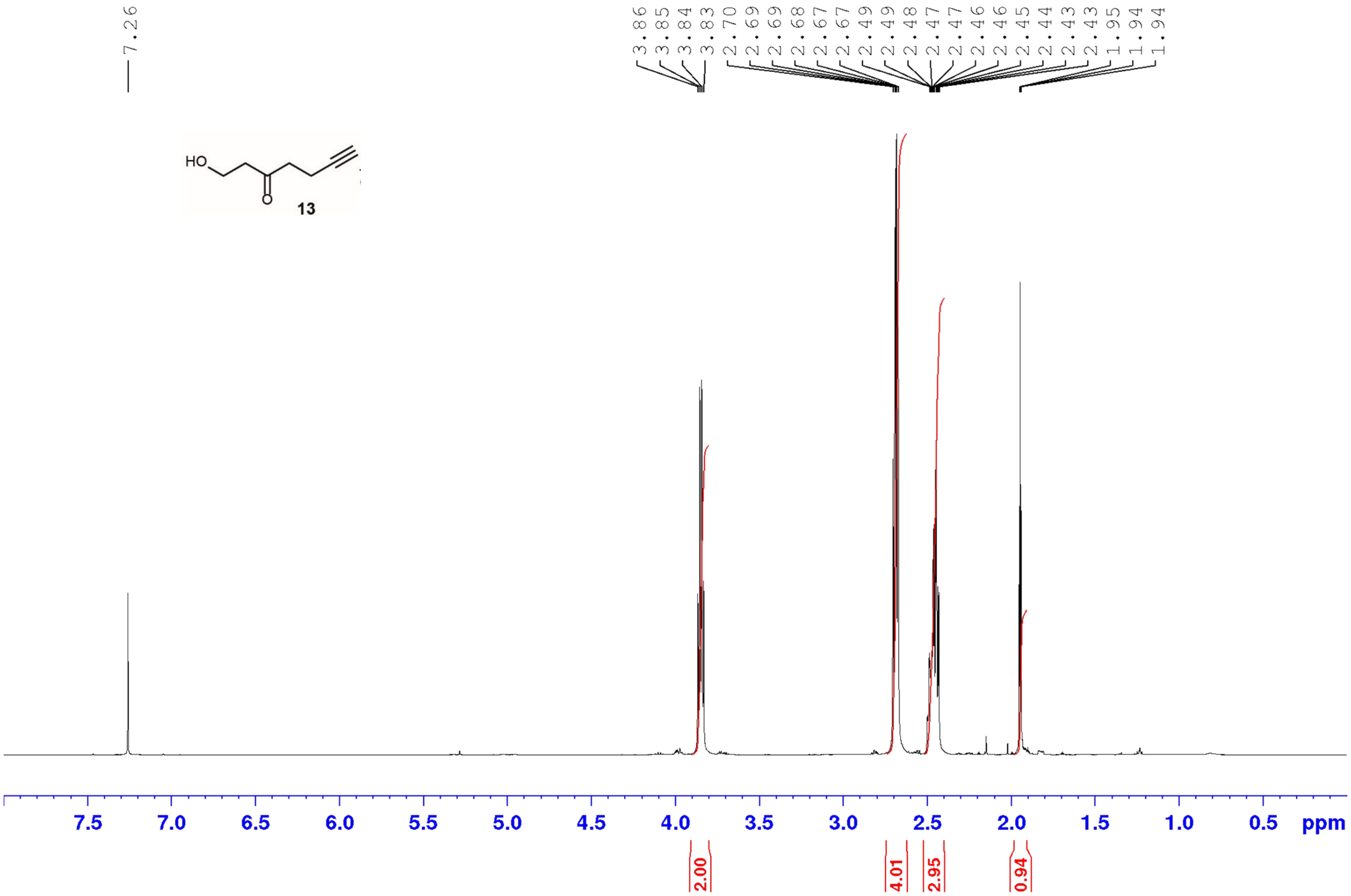

^{1}H NMR of 2-(3-(but-3-yn-1-yl)-3H-diazirin-3-yl)ethanol (**14**)

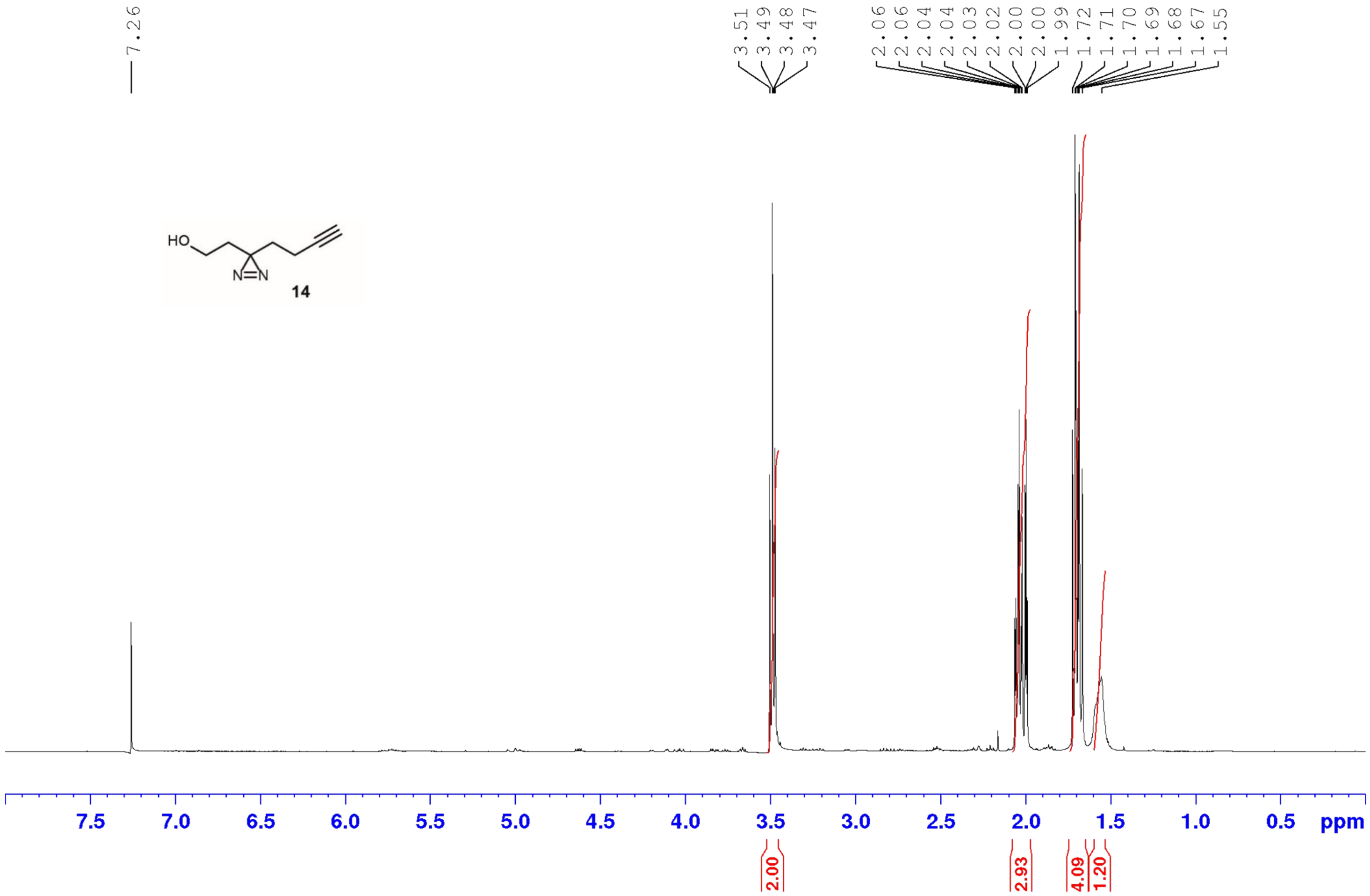

^{1}H NMR of 3-(but-3-yn-1-yl)-3-(2-iodoethyl)-3H-diazirine (**15**)

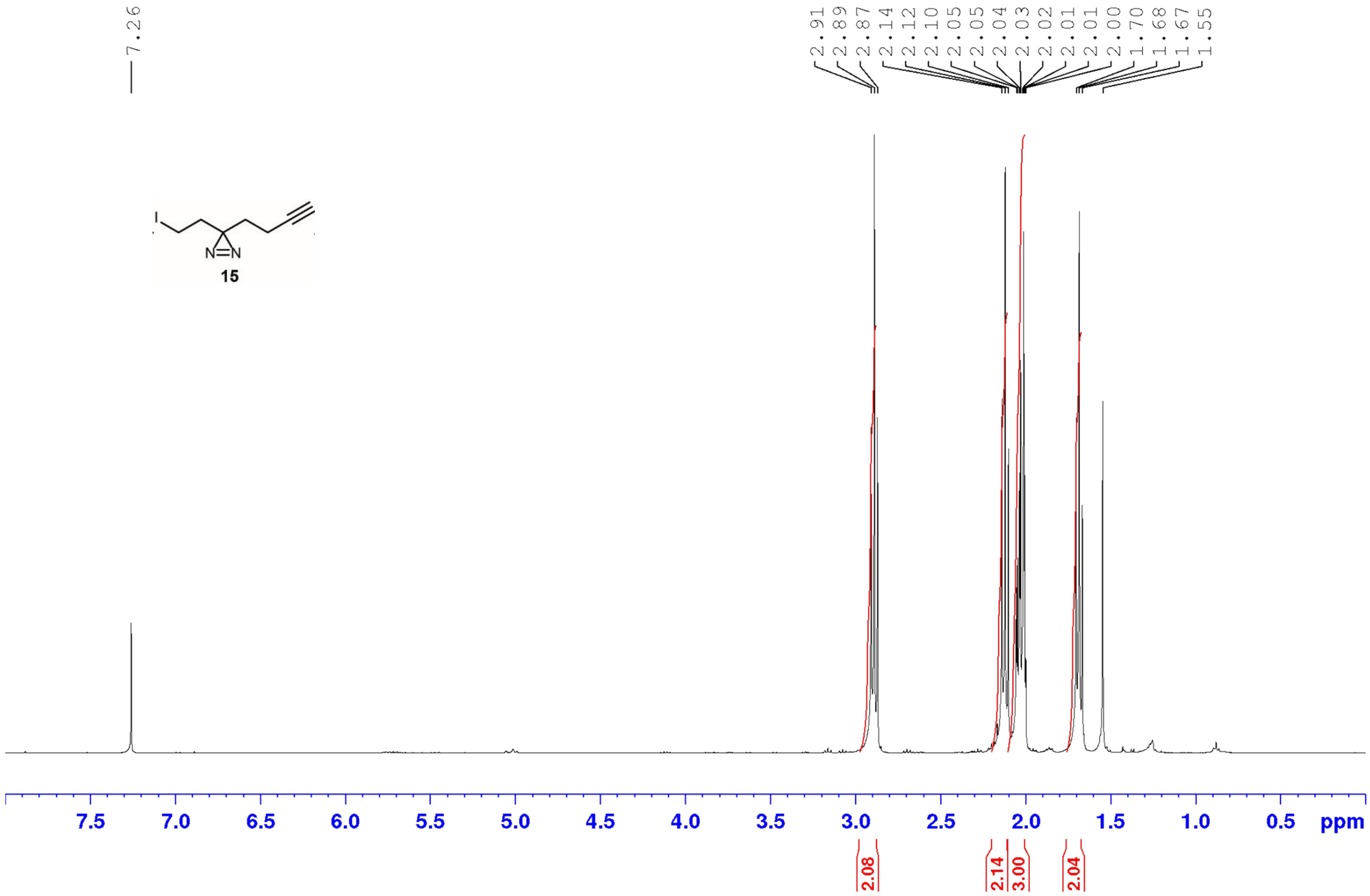

^{1}H NMR of *O*-methyl 2-(3-(but-3-yn-1-yl)-3H-diazirin-3-yl)ethanesulfono-thioate (**4**)

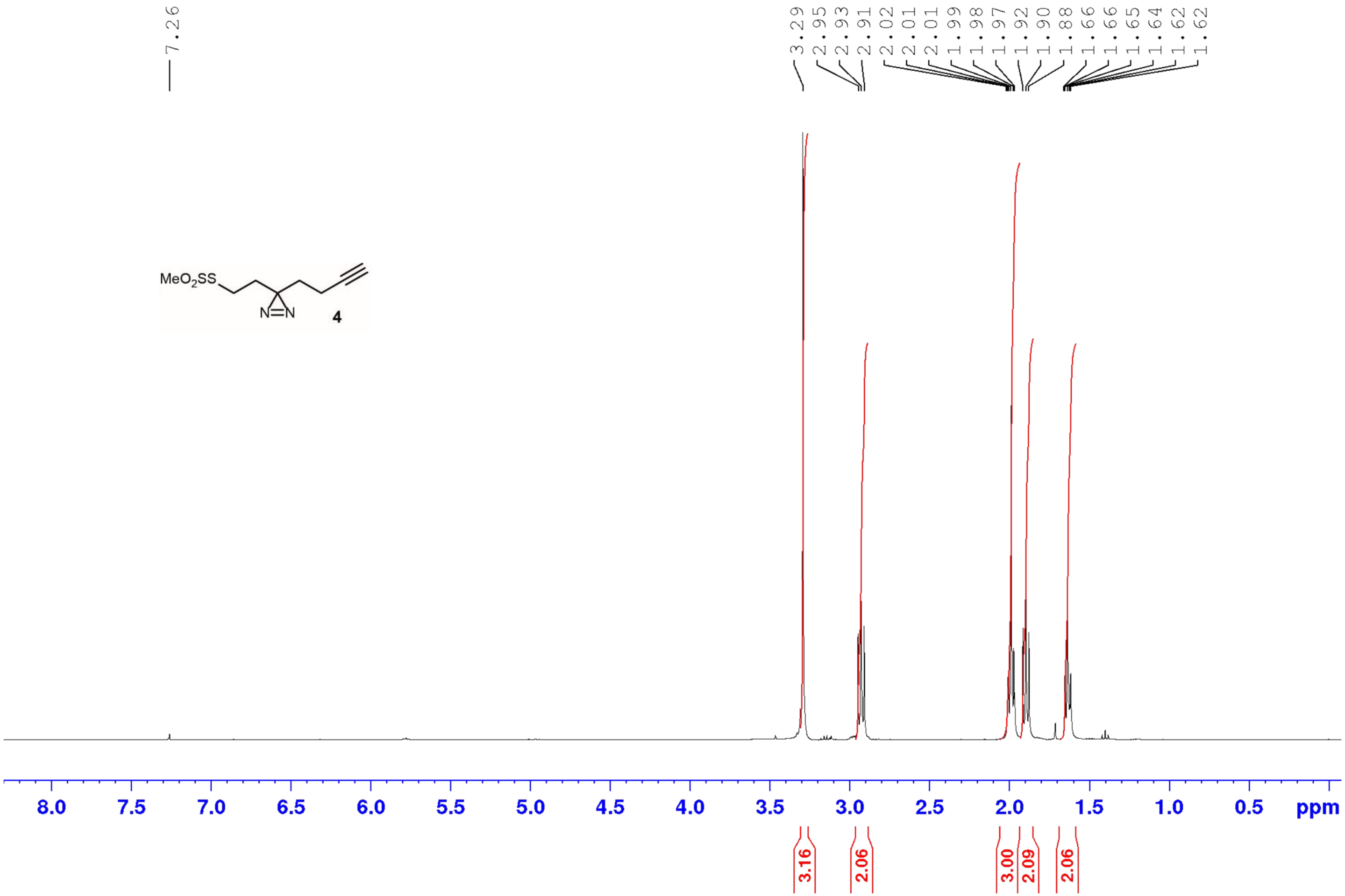

^{13}C NMR of *O*-methyl 2-(3-(but-3-yn-1-yl)-3H-diazirin-3-yl)ethanesulfono-thioate (**4**)

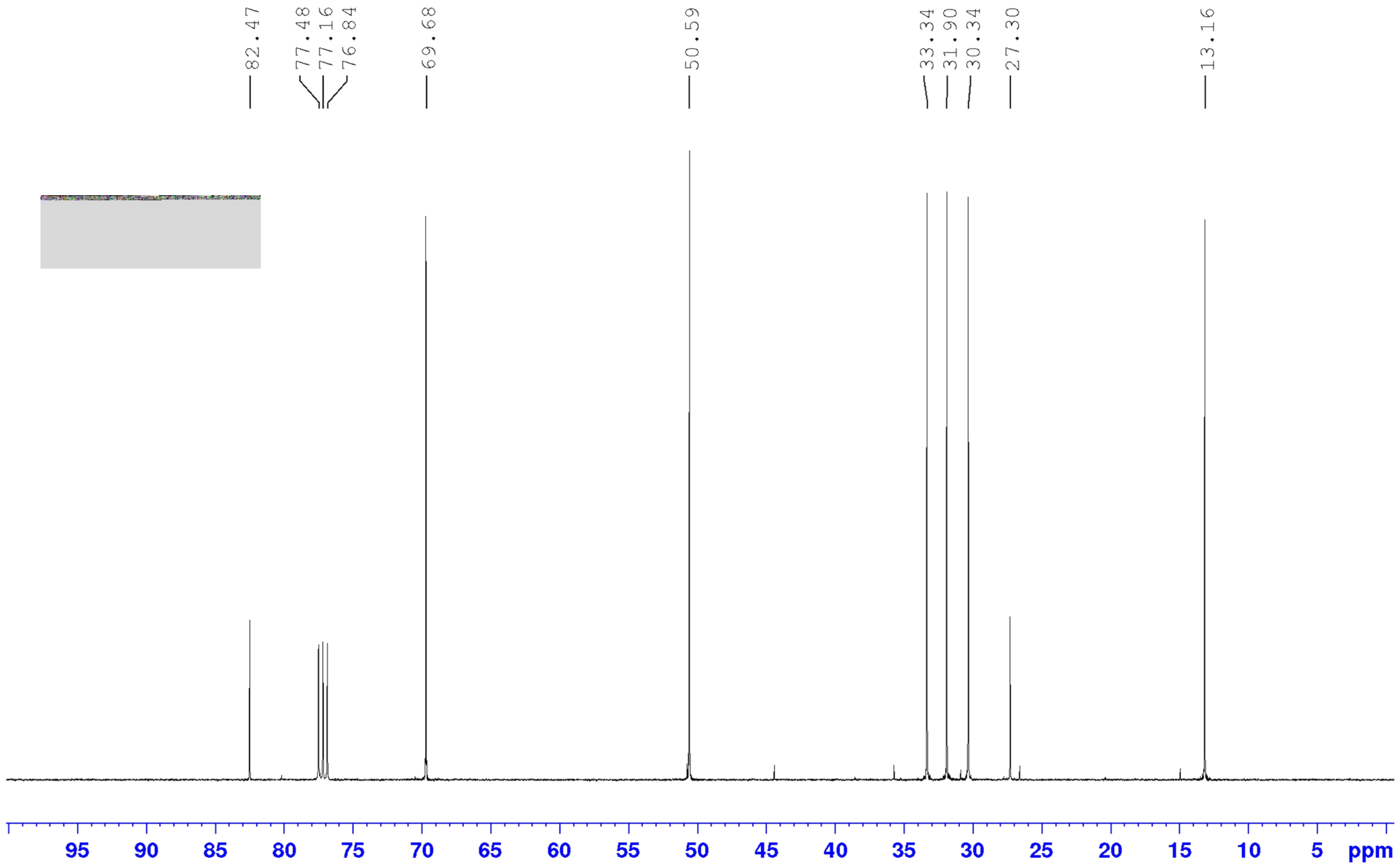

UV-vis of *O*-methyl 2-(3-(but-3-yn-1-yl)-3H-diazirin-3-yl)ethanesulfono-thioate (**4**)

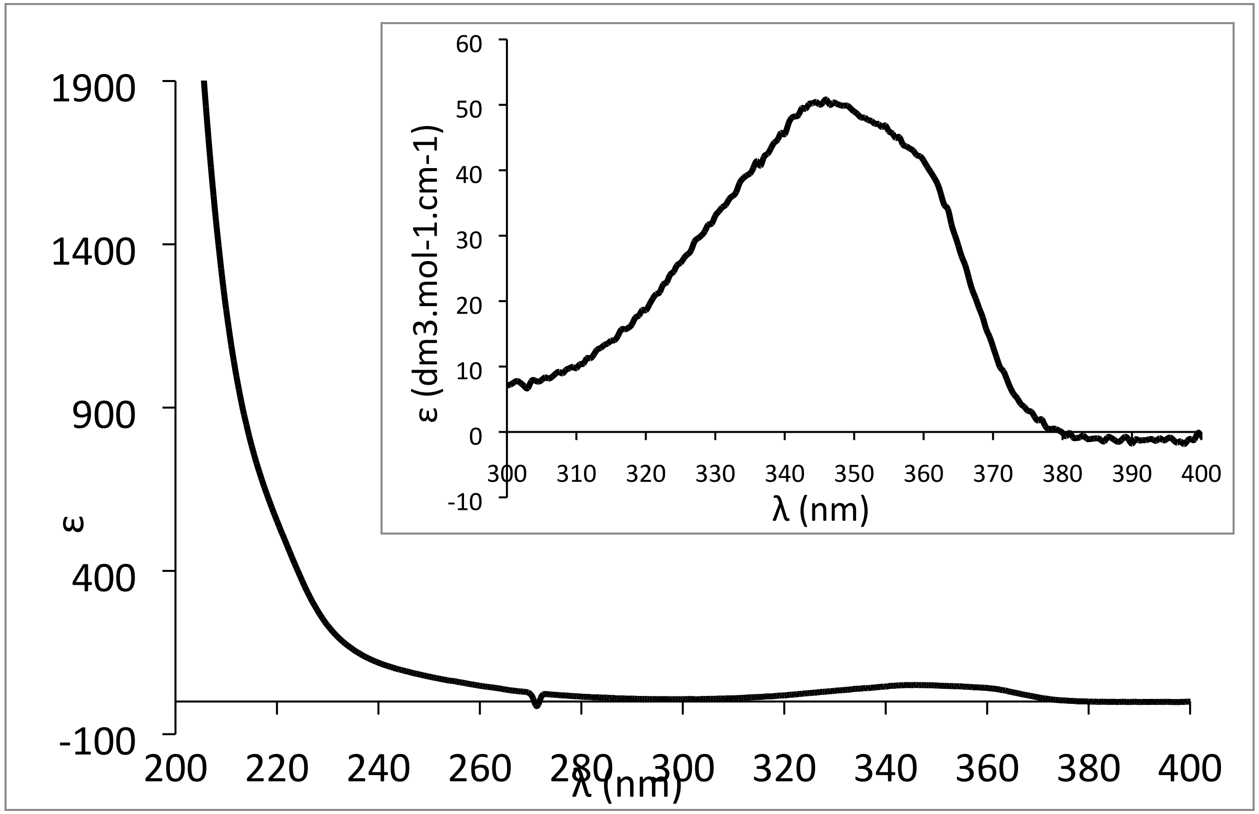

FT-IR of *O*-methyl 2-(3-(but-3-yn-1-yl)-3H-diazirin-3-yl)ethanesulfono-thioate (**4**)

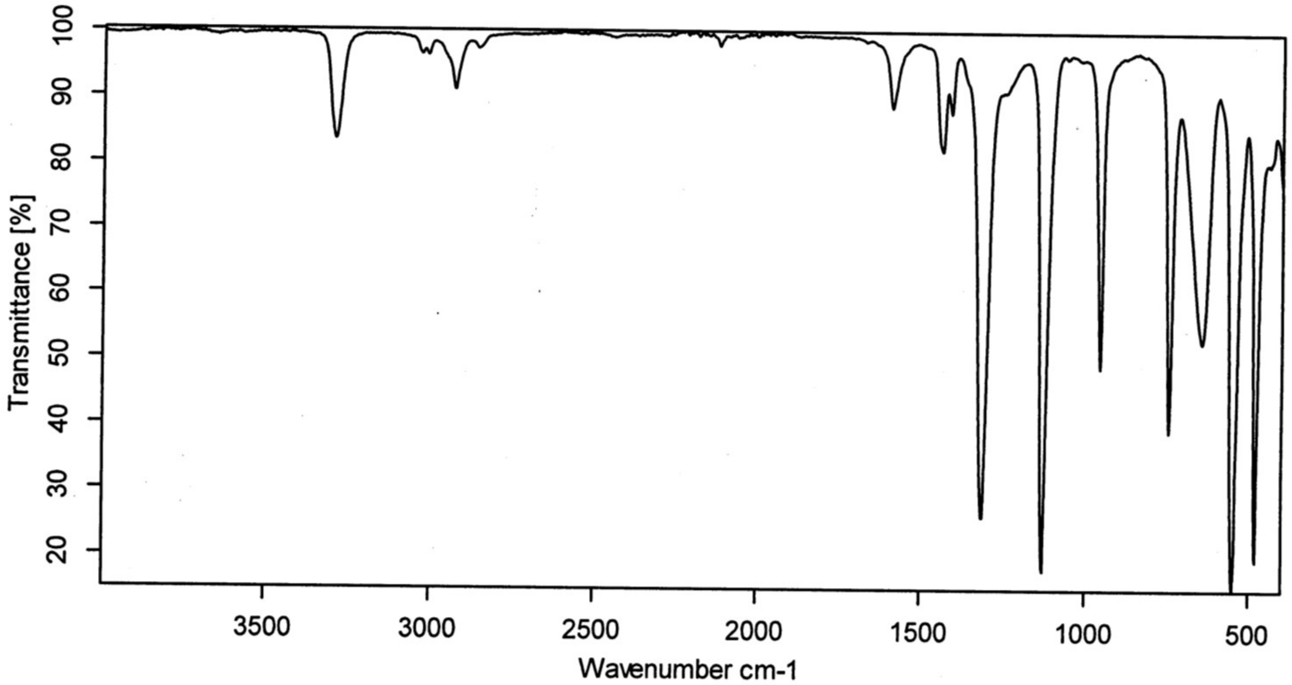

Supplement: Supplementary Information [file EMS205490-supplement-Supplementary_Information.pdf]
